# Morbidity Patterns in Primary Care in Hong Kong: Protocol for a Practice-Based Morbidity Survey

**DOI:** 10.2196/37334

**Published:** 2022-06-22

**Authors:** Julie Yun Chen, David Chao, Samuel Yeung-shan Wong, Tsui Yee Emily Tse, Eric Yuk Fai Wan, Joyce Pui Yan Tsang, Maria Kwan Wa Leung, Welchie Ko, Yim-chu Li, Catherine Chen, Wan Luk, Man-Chi Dao, Michelle Wong, Wing Mun Leung, Cindy Lo Kuen Lam

**Affiliations:** 1 Department of Family Medicine and Primary Care The University of Hong Kong Hong Kong China (Hong Kong); 2 The Hong Kong College of Family Physicians Hong Kong China (Hong Kong); 3 Department of Family Medicine & Primary Health Care Kowloon East Cluster Hospital Authority Hong Kong China (Hong Kong); 4 Division of Family Medicine and Primary Healthcare Jockey Club School of Public Health and Primary Care The Chinese University of Hong Kong Hong Kong China (Hong Kong); 5 Department of Family Medicine & Primary Health Care New Territories East Cluster Hospital Authority Hong Kong China (Hong Kong); 6 Department of Family Medicine & Primary Health Care Hong Kong West Cluster Hospital Authority Hong Kong China (Hong Kong); 7 Department of Family Medicine & Primary Health Care Kowloon Central Cluster Hospital Authority Hong Kong China (Hong Kong); 8 Department of Family Medicine & Primary Health Care Kowloon West Cluster Hospital Authority Hong Kong China (Hong Kong); 9 Department of Family Medicine & Primary Health Care Hong Kong East Cluster Hospital Authority Hong Kong China (Hong Kong)

**Keywords:** morbidity survey, primary care, general practice, family medicine

## Abstract

**Background:**

Up-to-date and accurate information about the health problems encountered by primary care doctors is essential to understanding the morbidity pattern of the community to better inform health care policy and practice. Morbidity surveys of doctors allow documentation of actual consultations, reflecting the patient’s reason for seeking care as well as the doctor’s diagnostic interpretation of the illness and management approach. Such surveys are particularly critical in the absence of a centralized primary care electronic medical record database.

**Objective:**

With the changing sociodemographic profile of the population and implementation of health care initiatives in the past 10 years, the aim of this study is to determine the morbidity and management patterns in Hong Kong primary care during a pandemic and compare the results with the last survey conducted in 2007-2008.

**Methods:**

This will be a prospective, practice-based survey of Hong Kong primary care doctors. Participants will be recruited by convenience and targeted sampling from both public and private sectors. Participating doctors will record the health problems and corresponding management activities for consecutive patient encounters during one designated week in each season of the year. Coding of health problems will follow the International Classification of Primary Care, Second Edition. Descriptive statistics will be used to calculate the prevalence of health problems and diseases as well as the rates of management activities (referral, investigation, prescription, preventive care). Nonlinear mixed effects models will assess the differences between the private and public sectors as well as factors associated with morbidity and management patterns in primary care.

**Results:**

The data collection will last from March 1, 2021, to August 31, 2022. As of April 2022, 176 doctor-weeks of data have been collected.

**Conclusions:**

The results will provide information about the health of the community and inform the planning and allocation of health care resources.

**Trial Registration:**

ClinicalTrials.gov NCT04736992; https://clinicaltrials.gov/ct2/show/NCT04736992

**International Registered Report Identifier (IRRID):**

DERR1-10.2196/37334

## Introduction

Primary care is the setting where the majority of people will seek health care. Studies on the ecology of health care show this is the case in the United States, where 52% of patients who consulted a doctor in the past month saw a primary care doctor [1], and also in Hong Kong (84.5% of patients) [2]. Information about the health problems of patients presenting in primary care is therefore crucial to understanding the health of the general community for the planning and allocation of health care resources.

It is important to replicate morbidity surveys from time to time given changing demographic and socioeconomic profiles. In Hong Kong, the last morbidity study was carried out in 2007-2008 [3]. Within the last 10 years (2005-2015), there has been an increase in the proportion of people aged 65 years or older, level of educational attainment, individual and household income, and cross-border immigration from mainland China, while there has been a notable decline in unemployment [4]. These sociodemographic changes are reflected in the health problems and concerns of patients who present to primary care and also in the changing prevalence of various diseases over time [5,6]. Practice-based morbidity surveys conducted at 10-year intervals have been able to capture changes in the prevalence and trends of health problems presenting in primary care as observed in England and Wales (1991-2001) [6] and in Hong Kong (1994-2007) [3,7].

Superimposed on this background is the expectation that chronic disease and psychological problems will continue to be a major burden of illness globally and in Hong Kong as we move deeper into the 21st century [8]. There is increasing pressure for primary care to shoulder the care for the majority of these patients given the volume of patients and the demonstrated effectiveness and cost-effectiveness of family doctor–led primary care [9]. Local Hong Kong evidence shows that chronic disease makes up an increasing proportion of problems seen by primary care doctors, with the bulk of patients seen in public outpatient clinics [10].

Since the last morbidity study in Hong Kong in 2007-2008 [3], the Hong Kong Government has implemented some primary care initiatives in response to the increasing burden on public general outpatient clinics (GOPC) such as the GOPC Public-Private Partnership, Elderly Health Care Voucher Scheme, and the Vaccination Subsidy Scheme. These aim to encourage patients to seek care in the private sector, particularly for care of chronic diseases. Tracking morbidity and management trends would provide an indication of the impact of these initiatives on the prevalence of chronic disease and its care in both public and private primary care sectors.

Apart from the morbidity pattern, specific information on the management pattern such as prescription, investigation, referral to specialists, health promotion, and disease prevention is important for assuring the quality of primary care services and for identifying support services needed. In 2014-2015, there were an estimated 6 million primary care encounters in the public sector alone [11], which extrapolates to about 30 million primary care encounters in the combined public (20% of total encounters) and private sectors across Hong Kong [12]. The most recent morbidity study found that the Hong Kong referral rate to specialists was 2.5% in 2007-2008 [3], which means that even a small increase of 1% in referral rate would produce a huge relative increase of 40%, translating to an additional 300,000 specialist referrals. Changes in consultation trends in primary care do occur [13], with substantial potential impact on health care service needs and expenditures, and must be monitored.

With an aging population, increasing prevalence of chronic disease, and the implementation of primary care initiatives to shift some of the burden of chronic disease care from the public to the private sector, a primary care morbidity study is timely and important to document and evaluate the impact of these changes.

This study aims to determine the changes in morbidity and management patterns in Hong Kong primary care compared with the patterns identified in the morbidity study conducted in 2007-2008 [3]. Specific objectives include the following:

1. To identify and describe the patterns of morbidity in primary care
2. To determine the management patterns of primary care doctors including prescription, investigation, referral, and preventive care
3. To examine the differences in morbidity patterns in public and private health care sectors
4. To determine the doctor and practice characteristics associated with morbidity and management patterns in primary care

## Methods

### Study Design

This is a prospective practice-based survey of Hong Kong primary care doctors that will explore the morbidity and management patterns in primary care to inform health care service and practice.

### Subjects

The target population in this study is primary care doctors in Hong Kong. We will invite doctors practicing in different primary care settings and in different regions of Hong Kong to join the study, reflecting the range of problems that may be characteristic of demographically different settings. We will include doctors working in the public (GOPCs and Department of Health clinics) and private sectors (solo practices, university health services, nonprofit organizations, and private hospital family medicine clinics).

### Sample Size Calculation

The sample size calculation is based on the first two primary objectives of our study, which are to determine the proportion of health problems/diagnoses among all clinical encounters and the proportion of various management activities in terms of prescription, investigation, referral, and preventive care. The 2007-2008 morbidity study found that respiratory conditions comprised the largest proportion of problems (36.2%) and among these, upper respiratory tract infection was the most common diagnosis (26.4%) [10]. On this basis, if we conservatively anticipate the proportion of a type of health problem/diagnosis to be 50% with a confidence interval of 95% and a maximum error of 0.5%, we will need to encounter at least 38,415 health problems over one year. This translates to about 10,000 health problems per season. In the 2007-2008 morbidity study, 109 doctors recorded 69,973 health problems over the course of the year, which meant that on average, each doctor encountered about 250 health problems in one week. Based on this estimation, 40 doctor-weeks will be required to encounter the requisite number of health problems for each season. Assuming there is a one-third dropout rate of doctors, 60 doctor-weeks per season will be required. According to a general population study on service utilization patterns in primary care, around 80% of those who visit a Western medical practitioner would attend a private clinic whereas the remaining 20% would attend publicly funded primary care services [12]. Considering this, we need 48 doctors from the private sector and 12 doctors from the public sector to participate in the study.

### Recruitment of Subjects

Because there is no complete, publicly available primary care doctor register with contact information, we will undertake a targeted recruitment. We will recruit doctors in the Primary Care Research Network established during a previous study on the epidemiology of depression in primary care conducted by the Department of Family Medicine and Primary Care at The University of Hong Kong. This resulted in a network of 60 primary care doctors across 45 practices who expressed interest to participate in future research studies [14]. In addition, we will invite members of the Hong Kong College of Family Physicians, honorary teachers of the Department of Family Medicine and Primary Care at The University of Hong Kong and the Division of Family Medicine, School of Public Health at the Chinese University of Hong Kong. We will also specifically target doctors listed in the Primary Care Directory, a voluntary directory, and the doctors listed as service providers in the GOPC Public-Private Partnership Programme on the corresponding websites. Those who are interested will receive a briefing on the study by the principal investigator and/or a member of the research team and provide written informed consent if they wish to participate.

### Data Collection

Data collection will take place for one week in each month of the year for 18 months starting March 2021, resulting in 18 designated weeks of data collection. This will permit evaluation of the variation in morbidity through the fifth and most serious wave of the COVID-19 pandemic in Hong Kong and across seasons, which has been a significant consideration in previous studies. Seasons may be divided into 3-month periods based on historical climatological data with spring as March-May, summer as June-August, autumn as September-November, and winter as December-February. Thus, there will be 3 weeks of data collection in each season. The study will run over 18 months to capture temporal trends as well as seasonal variation of diseases such as influenza. The first full week of each month in which there are no public holidays will be designated as the data collection week to maximize the number of working days. The week will be defined as 7 consecutive days from Sunday-Saturday as most private doctors in Hong Kong work on weekends and public GOPC are open for a half-day on Saturday. Each participating doctor will collect data on consecutive patients during any of the designated weeks of each season for a total of 6 weeks of data collected per doctor. The research team will collect all completed forms after each data collection week. All data will be checked for proper coding and be doubly entered, cleaned, and compiled into a central database for analysis.

### Data Collection Forms and Tools

#### Background Information Form

Each doctor participating in the study will provide background information including demographics, practice characteristics, training, and involvement in government-coordinated primary care initiatives. To maintain anonymity, doctors will be allocated a unique identifier to link their background information with the patient encounter data collected.

#### Encounter Form

Doctors will manually record information on every patient encounter that occurs during the designated data collection week on a standard data collection form. The information on this form includes basic patient demographics (age and sex), whether he/she is a new patient, the presenting problem as stated by the patient, the diagnosis (or corresponding International Classification of Primary Care, Second Edition [ICPC-2] code) as determined by the doctor, the management activities undertaken, drugs prescribed, and preventive care interventions initiated. Doctors can complete most of the form with checkmarks or simple abbreviations and can write in the diagnosis/problem if they are not sure of the ICPC coding. Doctors in the public sector GOPC are already using ICPC coding in their usual practice and may find it routine to record diagnoses in this way. For forms completed with written diagnoses, trained research assistants will translate these to the corresponding ICPC codes.

#### ICPC-2

ICPC-2 is the most widely used international classification for systematically capturing and ordering clinical information in primary care [15]. The ICPC-2 is widely used in general practice for the classification of three important elements of the health care encounter: reasons for encounters, diagnoses or problems, and processes of care.

### Outcome Measures

#### Primary Outcomes

1. Prevalence of health problems and diseases presenting in primary care
2. Rates of management activities including referral, investigation, prescription, and preventive care intervention by primary care doctors
3. Distribution of health problems and diseases presenting in the public compared with the private primary care setting
4. Factors that affect the proportion and distribution of health problems in the public sector compared with the private sector

#### Secondary Outcomes

1. Prescription rates of specific classes of drugs
2. Seasonal variation in primary care morbidity pattern
3. Relationship between patient’s presenting problem and doctor’s diagnosis

#### Confounding Variables

1. Demographic information of patients
2. Practice sector
3. Nature of the health problem (acute or chronic)
4. Background characteristics of the doctor and the practice

### Data Analysis

The patterns of morbidity in primary care will be presented by descriptive statistics for the prevalence of health problems and diseases, as well as the rates of management activities (referral, investigation, prescription, preventive care), weighted for the proportion of private and public primary care doctors in Hong Kong. The pattern of morbidity will be compared with the previous pattern in 2007-2008. All estimates will be accompanied by 95% CIs.

Stratified estimates in the patterns of morbidity in primary care by public and private doctors will also be reported and compared with the previous pattern in 2007-2008. The influence of the health care sector on morbidity patterns will be assessed by comparing the prevalence and rates of public and private doctors by nonlinear mixed effects analysis with logit link that accounts for potential covariance among health problems from the same doctor.

Factors associated with morbidity and management patterns in primary care will be assessed by nonlinear mixed effects model with logit link to cater for the dichotomous outcomes. Potential factors including patient demographics (age, sex), doctor background characteristics (age, qualifications, years of practice, type of practice, average number of consultations, participation in primary care initiatives), and nature of problem (acute, chronic, preventive) will be taken as covariates in the model. Both univariable and multivariable analyses will be performed. In the multivariable analysis, the presence of multicollinearity will be assessed by examining the tolerance. Factors involved in multicollinearity will only be considered one at a time.

### Ethical Approval

This study has received ethical approval from the Institutional Review Board of the University of Hong Kong/Hospital Authority Hong Kong West Cluster (UW 19-806), Hong Kong East Cluster (HKECREC-2021-091), Kowloon East Cluster (REC(KC/KE)-21-0124/ER-2), Kowloon Central Cluster (REC(KC/KE)-21-0131/ER-2), Kowloon West Cluster (161-01), and New Territories East Cluster (2021.368).

## Results

Data collection started March 1, 2021. As of April 2022, 176 doctor-weeks of data have been collected. The data collection will continue until August 31, 2022.

## Discussion

### Overview

In determining the approach for this study, we reviewed different data collection methods for morbidity and clinical practice in primary care to support the conceptualization and methodology selected. Commonly used approaches in gathering health and health service data in primary care include population surveys, retrospective analysis on medical records or registers, and practice-based morbidity surveys using patient encounter forms. However, methodological or practical considerations may limit the usefulness of any of these methods.

Population surveys such as general household surveys about individual health conditions and health service usage provide a broad-based perspective, where health problems that have not been consulted can also be identified [16]. However, the reporting relies on patient recollection [17], and the accuracy of morbidity identification largely depends on patient’s knowledge. Underreporting is also expected, especially for “sensitive” diseases such as sexually transmitted infections and cancer [16], or among older adult patients [18].

Data drawn from electronic medical records or registers of patient health care information provide rich databases for evaluating disease prevalence and morbidity trends in primary care as demonstrated in various international studies [19-24]. The data obtained are mostly accurate for medically diagnosed health problems. However, in the absence of centralized computerized databases, data retrieval may be difficult and labor intensive [25]. This is the case in Hong Kong, where centralized systems capture data only for the public government GOPCs, which provide only 20% of primary care.

On the other hand, practice-based morbidity surveys are a feasible option for capturing accurate and more comprehensive data [25]. They allow documentation of actual consultations, reflecting the patient’s reason for seeking care and the doctor’s diagnostic interpretation of the illness, which can be standardized by validated tools [16]. Both of these features are essential in health care service planning, which must rely on accurate information about health problems but also consider patient need. Because of this, such surveys continue to be used on a large scale in the United States, where the Centers for Disease Control and Prevention initiated the National Ambulatory Medical Care Survey in 1973 and has been running it annually since 1989 [26]. In Australia, the Bettering the Evaluation and Care of Health program collected general practitioner–patient encounter data for 18 years until 2016. These data were used widely for policy development and to improve Australian health care delivery and patient care [27]. Practice-based morbidity studies conducted in primary care settings have proven (and continue) to be a useful and reliable methodology for documenting disease prevalence and for service planning [6,10,28-32].

An issue that we encountered in the sample size calculation was whether we could assume that the number of health problems needed to encounter in each season should be evenly distributed. Such an assumption may not necessarily be reasonable, particularly for some season-specific diseases. However, based on the raw data of the earlier Hong Kong morbidity study, the overall most commonly occurring diagnosis was upper respiratory infection (26.4% of encounters), which was also the most commonly occurring diagnosis in all 4 seasons, ranging in frequency from 32.1% in winter to 22.7% in spring. The 14 diagnoses that account for >1% of visits were the same top 14 diagnoses across all 4 seasons and there was only variation in the order of frequency in each season. This is consistent with data from a study on the seasonal variation in diagnoses in primary care based on the United States National Ambulatory Medical Care Survey [33]. The key finding was that there was little seasonal variation among the 23 most commonly occurring diagnoses (which accounted for >1% of encounters)—only the rank order of the diagnoses varied from season to season.

As our sample size calculation is based on the primary objective of the study, which is to determine the prevalence of health problems/diagnoses among all clinical encounters, the calculation is derived from the proportion of the most commonly occurring diagnosis encountered in the earlier morbidity study. Since the most commonly occurring diagnosis accounts for 22.7%-32.1% of encounters in any given season, the conservative estimate of 50% that we used in our sample size calculation is reasonable to calculate the total number of health problems needed to encounter per season.

### Limitations

This study was originally planned and approved prior to the start of the COVID-19 pandemic. We delayed the implementation in the hope that the pandemic would pass quickly but as this has not happened, we will proceed with the expectation that the effect of COVID-19 will be a limitation of the study as it is affecting usual patient health care–seeking behaviors including for chronic disease [34]. We will also seize the opportunity to capture the full period of the worst (fifth) wave of the COVID-19 pandemic in Hong Kong and to examine how stringent pandemic control measures by the Hong Kong Government affect primary care consultations, and presumably the patterns after the surge has eased and things have returned back to normal.

## Appendix

Multimedia Appendix 1 Peer-review report by the Health and Medical Research Grant (HMRF) - Research Fund Secretariat, Research Office, Food and Health Bureau of Hong Kong.

## Abbreviations

GOPC: general outpatient clinic
ICPC-2: International Classification of Primary Care, Second Edition

## Data Availability

Data sharing is not applicable to this article as no complete data sets were generated or analyzed at this stage of the study.
