# Supplementary material for: Morbidity Patterns in Primary Care in Hong Kong: Protocol for a Practice-Based Morbidity Survey
Source: JMIR Res Protoc. 2022 Jun 22;11(6):e37334. doi: 10.2196/37334 (PMC9260520; doi:10.2196/37334)
Supplement: Multimedia Appendix 1 [file resprot_v11i6e37334_app1.pdf]

醫療衞生研究基金
Health and Medical Research Fund

Research Fund Secretariat, Research Office, Food and Health Bureau
9th Floor, Rumsey Street Multi-storey Carpark Building, 2 Rumsey Street, Sheung Wan, Hong Kong
Tel: (852) 3150 8987 Fax: (852) 2102 2444 E-mail:rfs@fhb.gov.hk

RESEARCH COUNCIL

Chairperson

Secretary for Food and Health / Permanent Secretary for Food and Health (Health)
食物及衞生局局長／食物及衞生局常任秘書長(衞生)

Members

Ms Mabel CHAU 周敏姬女士

Dr Vincent CHENG 鄭智聰醫生

Prof Annie CHEUNG 張雅賢教授

Prof David HUI 許樹昌教授

Prof Timothy KWOK 郭志銳教授

Prof LAU Chak-sing 劉澤星教授

Prof Diana LEE 李子芬教授

Prof LEUNG Suet-yi 梁雪兒教授

Prof LYU Aiping 呂愛平教授

Prof Alex MOLASIOTIS 莫禮士教授

Prof Hextan NGAN 顏婉嫦教授

Prof Malik PEIRIS 裴偉士教授

Dr Gene TSOI 蔡惠宏醫生

Prof YEOH Eng-kiong 楊永強教授

Prof YIP Shea-ping 葉社平教授

Dr YU Wai-cho 余衛祖醫生

Secretary for Innovation and Technology or representative
創新及科技局局長或代表

Director of Health or representative
衞生署署長或代表

Chief Executive of Hospital Authority or representative
醫院管理局行政總裁或代表

Dean of the Faculty of Medicine of The Chinese University of Hong Kong or representative
香港中文大學醫學院院長或代表

Dean of the Li Ka Shing Faculty of Medicine of The University of Hong Kong or representative
香港大學李嘉誠醫學院院長或代表

Our Ref.: FHB/H/41/153
Your Ref.:

Tel: 3150 8980
Fax: 2102 2444

30 November 2018

Dr Julie CHEN Yun
Associate Professor (Teaching)
Department of Family Medicine and Primary Care
The University of Hong Kong
3/F, Ap Lei Chau Clinic
161 Main Street, Ap Lei Chau, Hong Kong

Dear Dr CHEN,

Health and Medical Research Fund
Application for Research Grant (Ref: 16172011)
Application Round: December 2017

Thank you very much for your submission to the 2017 application round of the Health and Medical Research Fund (HMRF).

After careful consideration, I write to inform you that the Research Council considered your application titled "*A prospective practice-based study of the patterns of health problems, diagnoses and management in Hong Kong primary care*" as not supported.

Comments made by the Grant Review Board and expert referees, if any, are enclosed for your reference.

Your interest in applying to the HMRF is appreciated.

Please note that the Research Council's decision is final.

Yours sincerely,

Edmond Ma

Dr Edmond MA
Consultant (Research Office)
Food and Health Bureau

Encls.

cc *Research Services, HKU – Enclosures not attached.*

Research Council
Grant Review Board

GRB Assessment Report – Grant Application

Project No. : **16172011**
Project Title : **A prospective practice-based study of the patterns of health problems, diagnoses and management in Hong Kong primary care**
Principal Applicant (PA) : **Dr Julie CHEN Yun**
Rating : **1. Not supported**

GRB comments
1. There are concerns about the originality and clinical impact of the study.

Reviewers' comments
- The Reviewers' comments are attached for information only. No action is required.

Action required by PA
- No action is required.

Grading for applications	Meaning
4<br>Recommended for support	Nil or very minor issues to address only
3<br>Recommended for support subject to clarifications/ amendments	Minor revision and clarification required for a successful delivery
2<br>Not recommended for support at present	Major revision required for significant improvement
1<br>Not supported	Minimal impact on research / flaw in methodology/ incomplete application/ out of scope of the fund

REFEREE'S ASSESSMENT FORM

Reference No.: 16172011

Project Title: A prospective practice-based study of the patterns of health problems, diagnoses and management in Hong Kong primary care

PART A: REFEREE'S DETAILED REMARKS ON THE INDIVIDUAL SECTIONS OF THE GRANT APPLICATION

1. **Originality and Impact** What is the importance of the proposed research in terms of its originality and potential impact in the area under study? Is the proposed work original and important and will it influence practice?

> The proposal is not particularly original especially since it is the repetition of the same process that was conducted 10 years earlier. However, there is still merit in doing periodic assessment of health care delivery characteristics to determine how physician care and services have changes over time. A very similar survey is conducted every 10 years in the US and is very helpful in evaluating health care provider practice trends and capacity. In addition, the data often is used by research networks for comparative purposes to determine if their network members are representative of primary care practices. So the data have utility even beyond that for directly assessing how primary health care is delivered. It is less clear to me how these data will be applied in Hong Kong, but there are models across the world to provide examples to researchers. What isn't specified is whether these data will be made available to researchers in general which would increase the value of the study.

2. **Research Questions, Aims and Hypotheses** How specific, clearly expressed and realistic are the research questions, aims and hypotheses?

> The aims and objectives are clearly stated but understate the actual value of the data. As noted above, these data would be useful to other practices for benchmarking/comparison purposes or to evaluate the representativeness of practices that might be aggregated into a research practice network.

3. **Subjects and Study Methodology** (i) Is the proposed design and methodology appropriate for the study? (ii) Are sample sizes clear, justified, adequate and realistic? (iii) Are any preliminary data available? (iv) How feasible is the proposed timeframe?

> The study design benefits from previous experience the same investigators have had with their 2007 study and draws heavily from studies in other nations that have been conducted. The same size appears to be adequate and the sampling over multiple season provides additional generalizability to the findings. There are no primary data available, but the same team conducted these measurements ten years earlier which provides confidence that the data instruments work and that the investigators are capable of carrying out the project. The previous success of the research team also has demonstrated that the work plan is feasible and can be executed in the time frame laid out in the proposal.

4. **Outcomes and Data Analysis** (i) Are the primary and secondary outcomes clearly defined? (ii) Have potential problems been anticipated and addressed? (iii) Is the statistical/analytical design appropriate and clearly explained?

> The primary and additional secondary outcomes are delineated in the proposal as well as confounding variables that will need to be considered in the analyses. The analyses will account for confounding as well as for clustering effects which is appropriate. Seasonal effects will be considered by stratifying the data sets by the time of the year. While there are few details about the exact analytic approach, the overall strategy appears appropriate. The investigators have considered the potential issues associated with seasonal differences in acute care cases and presented the proposed strategy noted above to mitigate this potential problem.

5. **Research Capability** Comment on (i) the research team's expertise and track record (incl. principal investigator / project team members / collaborators) and (ii) the existing facilities of the Institution where the research will be conducted.

> The research team has broad experience in health services research and also experience in conducting this work in the past. There are a number of collaborators, all at very minimal effort, which can be concerning because it gives the appearance that no one person is the driving force that will assure this study gets done on time and on budget. The research infrastructure and facilities also appear to be adequate based on the description provided and the current and previous projects produced by the research team.

6. **Budget** Is the request for research personnel, consumables, equipment and overall budget justified and reasonable? [For reference, 1 USD is equivalent to approximately 7.8 HKD]

> The research budget is modest (especially considering how much is invested in the National Ambulatory Medical Care Survey in the US!) with only token payments to providers who participate and very little additional infrastructure funding (for computers, for example). The staffing level is also lean particularly if providers are going to provide paper records of their data.

7. **Ethical and Safety Considerations** Is the proposed research ethically sound? Outline any safety or ethical issues that from the proposed research and comment on whether these have been adequately addressed in the proposal. Has ethical approval been sought?

The research is ethically sound and the investigators have indicated that the proposal has been submitted for human subjects review. Based on the data being collected, there are few risks to either the doctors or their patients.

8. **Overall Comments and Conclusion** It is always helpful for applicants to receive constructive feedback from reviewers. What are the specific strengths and weaknesses of this proposal? Please include a brief overall appraisal of the proposal focusing on any areas for improvement and the basis for your comments, e.g. awareness of other work in the field.

Strengths:

These data will provide what promises to be a representative sample of primary care practice delivery patterns and health management strategies.

Weaknesses:

While it never specifically states that the surveys will be written, the reference to doctors "manually record(ing)" information and "Doctors can complete most of the form with checkmarks or simple abbreviations and can write in the diagnosis/problem..." makes it appear that this will all be hand-written. Providing the data form in an electronic version that can be completed on a tablet or even offering doctors the option of paper or electronic would reduce the entering of data by the study team significantly and would also reduce the potential for entry errors.

REFEREE'S ASSESSMENT FORM

Reference No.: 16172011

Project Title: A prospective practice-based study of the patterns of health problems, diagnoses and management in Hong Kong primary care

PART A: REFEREE'S DETAILED REMARKS ON THE INDIVIDUAL SECTIONS OF THE GRANT APPLICATION

1. **Originality and Impact** What is the importance of the proposed research in terms of its originality and potential impact in the area under study? Is the proposed work original and important and will it influence practice?

> This study repeats a similar study 10 years previously and seeks to determine changes in morbidity and management patterns in Hong Kong primary care compared with patterns identified in the morbidity study conducted in 2007-08.

2. **Research Questions, Aims and Hypotheses** How specific, clearly expressed and realistic are the research questions, aims and hypotheses?

> The aims and objectives centre around patterns of morbidity, primary care activities related to this including prescriptions, investigations, referrals, and preventive care, changes in these patterns since the previous survey and the determinants of any change. The aims, questions and hypothesis are clearly stated.

3. **Subjects and Study Methodology** (i) Is the proposed design and methodology appropriate for the study? (ii) Are sample sizes clear, justified, adequate and realistic? (iii) Are any preliminary data available? (iv) How feasible is the proposed timeframe?

> The design is a prospective survey of primary care consultations: the applicants seek to recruit at least 72 private and 18 public doctors to record data from consultations during one week every month for a year. Recuitment will be from existing networks of doctors participating in primary care studies and members of the Hong Kong College of Family Physicians, honorary teachers of the Department of Family Medicine and Primary Care, HKU and the Division of Family Medicine, School of Public Health, CUHK. A stratified sample of doctors from each of the 18 districts in Hong Kong will be recruited based on their location of practice and be proportionate to population size in each district.Although this is presented as feasible the applicants need to show that the management of patients and the patients presenting are broadly representative of patterns of morbidity and healthcare in the wider population of primary care doctors and patients.

4. **Outcomes and Data Analysis** (i) Are the primary and secondary outcomes clearly defined? (ii) Have potential problems been anticipated and addressed? (iii) Is the statistical/analytical design appropriate and clearly explained?

> Primary and secondary outcomes are clear and appropriate.

5. **Research Capability** Comment on (i) the research team's expertise and track record (incl. principal investigator / project team members / collaborators) and (ii) the existing facilities of the Institution where the research will be conducted.

> This is a strong research team with demonstrable expertise and experience in this area.

6. **Budget** Is the request for research personnel, consumables, equipment and overall budget justified and reasonable? [For reference, 1 USD is equivalent to approximately 7.8 HKD]

> The budget seems appropriate for the nature of the study being proposed.

7. **Ethical and Safety Considerations** Is the proposed research ethically sound? Outline any safety or ethical issues that from the proposed research and comment on whether these have been adequately addressed in the proposal. Has ethical approval been sought?

> The study proposed has the appropriate ethical processes and safeguards.

8. **Overall Comments and Conclusion** It is always helpful for applicants to receive constructive feedback from reviewers. What are the specific strengths and weaknesses of this proposal? Please include a brief overall appraisal of the proposal focusing on any areas for improvement and the basis for your comments, e.g. awareness of other work in the field.

Strengths:

> This study seeks to answer an important question about changes in morbidity and management patterns in Hong Kong primary care compared with patterns identified in the morbidity study conducted in 2007-08. The design and methods are well described. The team are experienced in the area and methods and have the capability to conduct the study which will provide important information for consumers, professionals, commissioners and policymakers.

Weaknesses:

> None.

REFEREE'S ASSESSMENT FORM

Reference No.: 16172011

Project Title: A prospective practice-based study of the patterns of health problems, diagnoses and management in Hong Kong primary care

PART A: REFEREE'S DETAILED REMARKS ON THE INDIVIDUAL SECTIONS OF THE GRANT APPLICATION

1. **Originality and Impact** What is the importance of the proposed research in terms of its originality and potential impact in the area under study? Is the proposed work original and important and will it influence practice?

The proposed study is essentially a repetition of a previously conducted survey and lacks of originality. Centre for Health Protection has routine surveillance networks for communicable and non-communicable diseases in Hong Kong and Hong Kong public healthcare sector accounts for nearly 90% of all in-patient services. The proposed study needs to highlight what significant and additional data will be provided on top of the existing ones.

2. **Research Questions, Aims and Hypotheses** How specific, clearly expressed and realistic are the research questions, aims and hypotheses?

The objectives and hypotheses are generally well specified.

3. **Subjects and Study Methodology** (i) Is the proposed design and methodology appropriate for the study? (ii) Are sample sizes clear, justified, adequate and realistic? (iii) Are any preliminary data available? (iv) How feasible is the proposed timeframe?

A survey design is appropriate for the proposed study. However, the proposed sampling method is still a convenience sampling. Although it is claimed that a stratified sample of doctors will be recruited from each of the 18 districts in Hong Kong, it remains unclear if and how each doctor in each stratum will be recruited with the same chance. The representativeness of the study sample and hence the generalizability of the study findings are of questionable. Furthermore, there are flaws in the sample size estimation. First, the sample size has not been determined upon the hypotheses raised in the study. Second, the margin of error assumed (0.5%) can be conservative for morbidities with high prevalence but it can lead to unreliable estimates particular for those morbidities with low prevalence. Third, the sampling unit should be doctor in the study design but the adjustment made for the design effect is rather vague, particularly it is unclear what is the average cluster size assumed in the estimation and how 63 doctor-weeks is derived.

4. **Outcomes and Data Analysis** (i) Are the primary and secondary outcomes clearly defined? (ii) Have potential problems been anticipated and addressed? (iii) Is the statistical/analytical design appropriate and clearly explained?

The reliability and validity of the study findings can be very much depended on the completeness and accuracy of the patient encounter information provided by the participating doctors. Since some doctors may encounter over hundred of cases daily, there is a lack of procedures to ensure or check if all encountered cases have been accurately reported. The data analysis plan sounds reasonable. However, more details regarding the temporal comparisons on the morbidity and management should be provided.

5. **Research Capability** Comment on (i) the research team's expertise and track record (incl. principal investigator / project team members / collaborators) and (ii) the existing facilities of the Institution where the research will be conducted.

n/a

6. **Budget** Is the request for research personnel, consumables, equipment and overall budget justified and reasonable? [For reference, 1 USD is equivalent to approximately 7.8 HKD]

n/a

7. **Ethical and Safety Considerations** Is the proposed research ethically sound? Outline any safety or ethical issues that from the proposed research and comment on whether these have been adequately addressed in the proposal. Has ethical approval been sought?

No ethical and safety issue is noted.

8. **Overall Comments and Conclusion** It is always helpful for applicants to receive constructive feedback from reviewers. What are the specific strengths and weaknesses of this proposal? Please include a brief overall appraisal of the proposal focusing on any areas for improvement and the basis for your comments, e.g. awareness of other work in the field.

Strengths:

The team consists of applicants with strong background and tracked experiences for the proposed study.

Weaknesses:

Although the team made great efforts to address the comments in the previous application, some methodological concerns as detailed above remain. In particular, the representativeness of the sample and generalizability of the study findings and reliability of some estimates with low prevalence is of concern.

REFEREE'S ASSESSMENT FORM

Reference No.: 16172011

Project Title: A prospective practice-based study of the patterns of health problems, diagnoses and management in Hong Kong primary care

PART A: REFEREE'S DETAILED REMARKS ON THE INDIVIDUAL SECTIONS OF THE GRANT APPLICATION

1. **Originality and Impact** What is the importance of the proposed research in terms of its originality and potential impact in the area under study? Is the proposed work original and important and will it influence practice?

This study is aimed to determine the changes in morbidity and management patterns in Hong Kong primary care compared with the patterns identified in the morbidity study conducted in 2007-08. In particular, it is to test the differences in the morbidity patterns between the public and private healthcare sectors. The management patterns mentioned include prescription, investigation, referral and preventive care. Doctor and practice characteristics associated with the morbidity and management patterns are also explored.

The proposed study is not original in the sense that it is aimed to generating results for comparison with those reported in a similar study conducted in 2007-8. The research design and practice-based method of collecting data are commonly used in other studies elsewhere. In view of the stipulated aim of the proposed study, this would not be regarded as a main concern. The results of the proposed study are supposed to have influence on the current and future practice of primary health doctors. Nevertheless, there are a few concerns which may influence such potential (see below).

2. **Research Questions, Aims and Hypotheses** How specific, clearly expressed and realistic are the research questions, aims and hypotheses?

The main hypotheses set for this study are on the differences in the results between the proposed study and the previous study conducted in 2007-8. In view of the differences in the population growth, and the known changes in the demographic and the health service policies and provision, the testing of differences between the two sets of results seem to be too superficial. Rather, the research team should consider setting more specific hypotheses in terms of the aging population, management of chronic diseases by the public sector, and new government policies on the Elderly Health Care Voucher Scheme and Primary Care Reference Frameworks (PCRF) for diabetes mellitus and hypertension for setting the hypotheses. Similarly, the same approach can be applied to the hypothesized differences between the public and private primary health doctors. More theory-led and/or policy-specific hypotheses would result in stronger impact of the findings for driving changes in the doctor's practice and/or government policy development.

3. **Subjects and Study Methodology** (i) Is the proposed design and methodology appropriate for the study? (ii) Are sample sizes clear, justified, adequate and realistic? (iii) Are any preliminary data available? (iv) How feasible is the proposed timeframe?

It is not clear how the design and methodology described in this proposal similar with or different from those employed in the 2007-8 study. To allow meaningful comparisons between the two studies, the research design and methodology should be very similar if not identical. The use of stratified sampling in the proposal study needs to be further articulated in terms of the likelihood of generating different results from those of the previous study. The use of the same method for data collection also raises a concern as the results of the previous study has not been widely reviewed by local and international researchers. This comment is made in view of the fact that the results of the previous study have not been published in journals which can reach international readers.

The sample size calculation is based on testing the differences between the proposed and previous studies. However, it does not consider the heterogeneity of the participants to be recruited based on stratified sampling. For instance, for the participants in private practice, they will come from 18 districts. What it means is that there will be only four from each of the districts. It would therefore be difficult to test the potential differences among the district-based sub-sample. Besides, there could be differences in the management patterns due to group versus solo practice etc. The research team should justify how the sample size calculation based on the between-cohort differences can tackle the potential heterogeneity among the participants in both the private and public sector.

The employment of practice-based approach for collecting the data has its merit. Nevertheless, there should be measures to be employed to safeguard the validity of the data to be collected solely from the practicing doctors.

No preliminary data is available and the proposed timeframe is appropriate.

4. **Outcomes and Data Analysis** (i) Are the primary and secondary outcomes clearly defined? (ii) Have potential problems been anticipated and addressed? (iii) Is the statistical/analytical design appropriate and clearly explained?

The primary and secondary outcomes are clearly defined. The statistical analyses are appropriate.

5. **Research Capability** Comment on (i) the research team's expertise and track record (incl. principal investigator / project team members / collaborators) and (ii) the existing facilities of the Institution where the research will be conducted.

The research capacity particularly the co-investigators is good. They have the expertise and track record for supporting the proposed study.

6. **Budget** Is the request for research personnel, consumables, equipment and overall budget justified and reasonable? [For reference, 1 USD is equivalent to approximately 7.8 HKD]

The budget is reasonable.

7. **Ethical and Safety Considerations** Is the proposed research ethically sound? Outline any safety or ethical issues that from the proposed research and comment on whether these have been adequately addressed in the proposal. Has ethical approval been sought?

The proposed study is ethically sound.

8. **Overall Comments and Conclusion** It is always helpful for applicants to receive constructive feedback from reviewers. What are the specific strengths and weaknesses of this proposal? Please include a brief overall appraisal of the proposal focusing on any areas for improvement and the basis for your comments, e.g. awareness of other work in the field.

Strengths:

The strength of the proposed study is to compare the results to be generated to those reported in a previous study conducted 10 years ago. The use of practice-based approach for collecting data directly from the participating primary health doctors is a merit. The findings have the potential to influence practice of doctors, policy making in primary health, and education of doctors.

Weaknesses:

The proposed study should consider going beyond testing the differences between the two studies but asking demographic- and policy-specific questions. This would strengthen the impact of the new findings to be generated from the proposed study. The differences in the sampling and data collection method may hamper the meaningfulness of the between-study comparisons.
